# Toxicity of respirable coal dust: An overview of origin, chemistry, mechanisms, and possible remedies

**DOI:** 10.1007/s40789-025-00841-x

**Published:** 2026-01-05

**Authors:** Amir Eskanlou, Barbara J. Arnold

**Affiliations:** John and Willie Leone Family Department of Energy and Mineral Engineering, College of Earth and Mineral Science, The Pennsylvania State University, University Park, PA 16802 USA

**Keywords:** Quartz, Pyrite, Coal, Clay, Toxicity, Respirable dust

## Abstract

The prevalence of coal workers’ pneumoconiosis (CWP) has been on the rise among U.S. coal miners since the early 2000s and is most pronounced in central Appalachia. Such adverse effects on human health are partly linked to the toxicity of respirable coal mine dust particles. In this review, we present an overview of the characteristics and health effects of the coal dust components, as well as the origin, chemistry, and mechanisms for their potential toxicity. Toxicity of coal mine dust is linked to the surface chemistry and bioactivity of the composing particles, such as coal, crystalline silica (quartz), pyrite, and sometimes diesel particulate matter. Formation of reactive oxygen species, such as •OH on the surface of these particles, contributes to the toxicity of coal dust. Various mechanisms including the metal-micelle coating, polymer-coating, chelation of the surface metal ions, and surface silanization, have been proposed in previous studies for the reduction or inhibition of the toxicity of coal dust particles. However, due to the complexity in surface chemistry of the various minerals and coal, there is no single universal detoxification mechanism for coal dust. One feasible remedy to address the proposed mechanisms of toxicity is the use of chemical additives as wetting agents. Important considerations for this approach include particle size and aging, mineralogy, morphology, chemical composition, solubility, surface charge, and synergistic effects of the composing phases. Additional factors to consider are the solution quality and composition, coal rank, and the potential hazards and costs associated with the chosen chemical agents.

## Introduction

Coal is an important source of energy in the U.S. and globally. Coal mining is one of the oldest occupational activities still performed on a large scale. According to the Energy Information Administration (EIA), as of January 2022, the U.S. has 251 billion short tons of recoverable coal reserves, of which about 58% is underground mineable coal (EIA (Energy 2022)). In 2022, about 546 million short tons of coal were consumed in the U.S., with 91.9% being consumed for electric power generation (EIA (Energy 2021)). Apart from offering benefits, coal mining carries various health-related issues including exposure to noise, heat, and airborne dusts causing many associated health problems (Borm 2002). Workers in underground coal mines are at greater risk of occupational lung diseases than surface miners (Naghadehi et al. 2014). While nearly eliminated in the 1990 s, Coal Worker’s Pneumoconiosis (CWP, commonly known as “black lung”) was observed to increase in prevalence and severity in the early 2000 s especially in central Appalachia (Arnold 2016). Data from epidemiological studies and health surveillance reports indicate that coal miners in the Appalachian (West Virginia, Kentucky, Virginia, and Pennsylvania) and Interior regions are at a higher risk of CWP prevalence compared to the Western region of the U.S. (Arnold 2016; Attfield and Morring 1992; Blackley et al. 2018; Morgan 1968). Similar findings indicating an increased prevalence of lung diseases in the Appalachian region have been obtained by the National Institute for Occupational Safety and Health (NIOSH) when examining chest radiographs from the Coal Workers’ Health Surveillance Program (CWHSP) (Salinas et al. 2022). According to the Centers for Disease Control and Prevention (CDC), between 2007 and 2016, CWP was identified as the primary or contributing factor in the deaths of 4,118 miners (NIOSH (The 2022)). Between 1970 and 2016, CWP was the primary or contributing factor in the deaths of a total of 75,178 miners (NIOSH (The 2022)). According to the Work-related Lung Disease Surveillance Report, CWP deaths in the U.S. accounted for half of all pneumoconiosis deaths during the 10-yr period between 1990 and 1999 (Zhang Qi, Huang, 2005). A similar increasing trend of CWP incidents has been reported in China, according to a study of patients over a time span of more than 50 years from 1949 to 2021 (Wang et al. 2023). Despite tremendous efforts to reduce miners’ exposure to coal dust, the associated lung diseases remain a major concern in the coal mining industry. The main goals have been to understand and describe the connections between coal dust exposure and its adverse effect on human health and to determine the possible biological mechanisms involved in these connections (Graber et al. 2017).

Coal has many components: carbon that changes with coalification or rank, crystalline silica (mainly quartz), pyrite, and other minerals, including calcite, dolomite, and clays (mostly kaolinite followed by chlorite) that may be associated in many ways in the coal (Sarver et al. 2021; Tishmack and Burns 2004). The particles generated from coal mining operations (coal dust) include fine particles of these components and, in some cases, diesel particulate matter (DPM) in mines that utilize diesel-powered equipment (Zhang et al. 2021). Such particles, due to their airborne respirable size (*d*_80_ < 10 μm, *d*_50_ ≤ 4 μm), can penetrate to terminal bronchioles and the lung gas-exchange regions, which can be the first step in developing various forms of lung diseases (Liu and Liu 2020; Prevention 1999). In addition to CWP, asthma, chronic obstructive pulmonary disease (COPD), silicosis, and cardiovascular diseases have been linked to prolonged exposure to respirable coal dust (NASEM (National 2018)). Therefore, coal dust has been a serious environmental and health issue in the coal industry posing irreversible harm to the health of coal mining workers especially in the underground areas with higher levels of exposure (Naghadehi et al. 2014; Scaggs 2016).

Particle size, morphology, mineralogy, solubility, surface area, coal rank, chemical composition, and surface properties of coal dusts are the major factors influencing the harmful effect of coal dust (Erol et al. 2013; Gautam et al. 2016). The toxicological effects of dust particles vary depending on the physicochemical properties within the dust mixture (Kumar et al. 2021; Zazouli et al. 2021), with a strong association between the chemical composition of respirable dust and the associated biological response, including the potential of transition metals like Fe to cause oxidative stress by inducing ROS generation (Rice-Evans et al. 1991; Rodrigo-Moreno et al. 2013; Wilson et al. 2002). Once generated and inhaled, the surface of these freshly cleaved particles undergoes oxidation within the human respiratory system, leading to the production of reactive oxygen species (ROS) like hydroxyl radicals (•OH), which are primarily responsible for the adverse health effects of coal dust causing oxidative stress in human cells (Kelly and Fussell 2017; Schins and Borm 1999; Zazouli et al. 2021). Inside the human lung, •OH diffused from the surface of dust particles attacks lung tissues to gain an electron, causing severe damage to the lung tissues (Cadet et al. 1999; Schins and Borm 1999), which can be described as dust toxicity (Kumar et al. 2021; Zazouli et al. 2021). Respirable coal dust also has a large surface area that enhances the surface reactivity and, therefore, the generation of ROS (Hussain et al. 2009; Kumar et al. 2021). Therefore, knowledge of the physicochemical characteristics, surface properties as well as the mechanisms of surface oxidation for the various coal dust components is essential in understanding the toxicological responses associated with coal dust.

This paper explores the surface chemistry properties, health effects, and potential remedies for the toxicity of coal dust components, with a focus on providing a critical review of current and emerging research practices. By compiling this information, the paper aims to enhance scientists’ and engineers’ understanding of these issues, ultimately contributing to the future development of biologically inert coatings for coal dust particles.

## Respirable dust

**Fig. 1:**
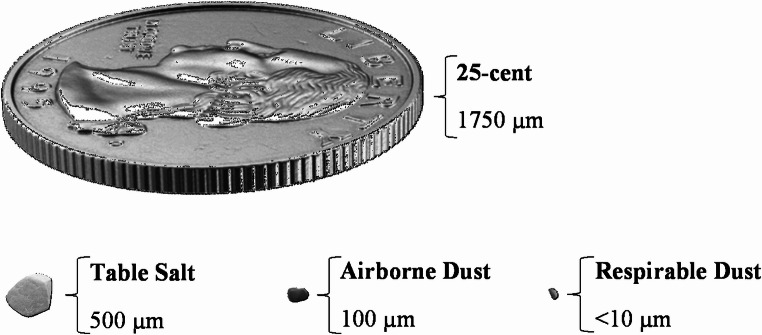
Dust particles compared to a table salt particle and a 25-cent piece

Respirable dust refers to the fraction of airborne particles with diameter less than 10 μm, which are small enough to penetrate to terminal bronchioles and the lung gas-exchange regions (Brown et al. 2013). Figure 1 compares the respirable and airborne dust particles to a table salt particle and a 25-cent piece. As respirable dust particles penetrate deep into the respiratory system, they pose serious health risks to humans (Donaldson et al. 2002). The NIOSH recommended exposure limits (REL), which are defined as the maximum concentration of dust that a worker can be exposed for up to a 10-hr workday during a 40-hr workweek, are 1 and 0.05 mg/m^3^ (time-weighted average concentration) for coal dust and quartz dust, respectively (Barsan 2007; Kuempel 1995). The mean concentration of respirable dust in the U.S. underground coal mines between 1982 and 2017 has been estimated to be 0.55 mg/m^3^, with the highest exposure in longwall and continuous miner operators (Doney et al. 2019). In the coal mines of Shanxi (China), workers other than those at the coal face were reported to be exposed to the highest level of coal dust (16.85 mg/m^3^) (Tong et al. 2019), indicating that workers in different areas of a mine can experience excessive exposure to respirable dust. A visual comparison of particulate matter among different workplaces has shown that the particles generated in transportation zones have the smallest diameters followed by coal face zones (Zazouli et al. 2021). The smaller the size of the respirable particles, the more harmful they are to human health due to their easy penetration into the respiratory system and their higher surface reactivity (Mischler et al. 2016). The surface chemistry properties of respirable coal dust are discussed in the following section.

## Surface chemistry properties of concern

The toxicity of respirable dust particles is primarily linked to their surface chemistry, and a significant contributor to their toxicity is their potential to induce the formation of ROS such as •OH. A free radical is broadly defined as a molecule or ion with an unpaired electron, making it highly reactive (Fubini and Fenoglio 2007). Oxygen is a significant source of radicals in biological systems due to its molecular structure, which allows it to accept free electrons from normal metabolic processes (Blake et al. 1987). This leads to the formation of ROS like the superoxide anion (O_2_^•−^), •OH, and hydrogen peroxide (H_2_O_2_) (Blake et al. 1987; Valencia et al. 2018). The generation of these ROS is influenced by the presence of oxygen, the redox state of mitochondrial complexes, and the mitochondrial membrane potential (Valencia et al. 2018). Various intracellular processes, such as the mitochondrial respiratory chain, have the capability to convert molecular oxygen into ROS (Blake et al. 1987). At low concentrations, these substances are only moderately reactive, and any potential harmful effects can be minimized by the human cell’s antioxidant defense mechanisms, such as superoxide dismutase, catalase, and glutathione peroxidase (Buonocore et al. 2010; Raes et al. 1987). However, when transition metal ions such as iron are present, these radical species, along with free oxygen, can produce •OH, a highly reactive free radical (Rodrigo-Moreno et al. 2013). Hydroxyl radicals interact with nucleic acids either by removing hydrogen from the sugar component or by adding to the bases. These interactions lead to the formation of new radical groups and the breakdown of the nucleic acid chains (de-polymerization) (Berndt et al. 2016; Cheeseman and Slater 1993; Cohn et al. 2006a). This can cause a range of harmful effects, including DNA damage, protein oxidation, and lipid peroxidation (Cadet et al. 1999). Unlike molecular toxicants, particle toxicants including respirable dust, interact with living organisms primarily at their surfaces (Zazouli et al. 2021). This means that the effects of particle toxicants may be determined by their surface chemistry. Therefore, understanding the surface chemistry of respirable dust is crucial for predicting its health effects, establishing safety standards, and designing protective measures (Fubini and Areán 1999; Kuhn and Demers 1992). In what follows, we will explore the effects of the major surface chemistry properties of respirable dust particles.

### Surface area

Respirable dust particles, especially those with diameters between 1 and 5 micrometers, due to their large surface area to volume ratio, can interact more readily with lung tissues and penetrate deep into the alveoli. This has implications for the toxicity and biological activity of dust particles. As the specific surface area of the dust is increased, the rate of oxidation and particle-induced •OH production is further enhanced due to higher surface reactivity (Chen et al. 2022; Schoonen et al. 2006). This leads to increased potential for inflammation, oxidative stress, and other toxic effects (Schoonen et al. 2006). Particles with larger surface areas have more sites available for adsorbing or binding other molecules/substances (Boers et al. 2005). This means that they can carry other toxicants or compounds from the environment into the respiratory system, potentially enhancing their toxicity (Fubini & Areán, 1999). The larger surface area of the inhaled dust particles can enhance the rate at which certain toxic components of the dust dissolve into the lung fluid (Giordani et al. 2022; Zhong et al. 2017). Moreover, particles with larger surface areas might be taken up by lung cells more readily, potentially leading to intracellular effects such as inflammation, cytotoxicity, and even genotoxicity (Boland et al. 1999; Churg 1996; Knaapen et al. 2002; Schins et al. 2002). Further discussion on these matters will follow. It should be noted that particles that are too large or too small may not be readily endocytosed.

### Surface functional groups

The presence of specific functional groups (e.g., hydroxyl, carbonyl) on the surface of dust particles can influence their reactivity and interaction with biological molecules (Jiang et al. 2023; Liu et al. 2021). For example, siloxyl radicals (SiO•) on a freshly cleaved quartz surface are believed to play a role in the generation of •OH through the reaction of the SiO• radicals with molecular oxygen to form O_2_^•−^ (Konecny et al. 2001; Pavan et al. 2020; Shi et al. 1995). This superoxide can then participate in various reactions to produce •OH. This matter is explored in more detail in Sect. 6 dedicated to the quartz-specific issues. The chemical properties of coal including its surface charge (that is, the presence of H^+^ or OH^−^ ions) and its ability to adsorb harmful gases are significantly influenced by its surface functional groups (e.g., carboxyl, pyridine) (Levine et al. 1982; Liu and Liu 2020), which can be determined by various methods such as Fourier transform infrared spectroscopy (FTIR), nuclear magnetic resonance (NMR), and X-ray photoelectron spectroscopy (XPS) (Miao et al. 2015; Wang et al. 2011; Xin et al. 2014).

### Metal content

Respirable dust can contain trace amounts of metals like As, Fe, Pb, Hg, and Se among others (Kazantzis 1981; Schroeder et al. 1987) that can leach from the dust particles when they contact biological fluids. Many of these metals can be toxic to cells and can lead to various health effects, including neurotoxicity and carcinogenesis (Rice-Evans et al. 1991; Rodrigo-Moreno et al. 2013; Wilson et al. 2002). Transition metal ions play a significant role in the biological effects of oxygenated free radicals (Rodrigo-Moreno et al. 2013). For instance, a Fenton type reaction (Cohen 1985), where H_2_O_2_ interacts with a transition metal ion (Rxn. 1), is typically a primary step in the formation of the free radicals:

1 $$\:\mathrm{M}^{n+}+\mathrm{H}_{2}\mathrm{O}_{2}\:\to\:\:\mathrm{M}^{\left(n+1\right)+}+\mathrm{OH}^{-}+\:{\bullet\:}\mathrm{O}\mathrm{H}$$

where M^n+^ is a metal ion in its “low oxidation” state, usually Fe^2+^, Ti^3+^, V^4+^, and Cr^5+^. The reduced metal ion species interact with H_2_O_2_ in a continuous cycle to produce •OH (Imlay et al. 1988). Therefore, even trace amounts of metal ions that are adsorbed to mineral dusts, like quartz, can catalyze the creation of the highly toxic free radicals from cellular hydrogen peroxide (Schoonen et al. 2006).

### Hydrophobicity

Solid substances with heteropolar surfaces are hydrophilic because the exposed surface ions strongly interact with the electric dipole of water molecules, while homopolar surfaces tend to be hydrophobic (Fubini and Areán, 1999). The water-attracting or repelling properties of dust particles influence their behavior in the lung fluid (Xu et al. 2017), while level of hydrophilicity affects how particles are cleared from the body (Fubini and Fenoglio 2007). Hydrophobic particles might not be cleared effectively from the lungs, leading to prolonged retention and increased potential for toxicity (Jing et al. 2010; Li et al. 2013; Wang et al., 2017). Mineral particles with hydrophilic properties exhibit strong interactions with biological molecules, primarily those that are polar or contain charged groups, enabling them to serve as electron donors or acceptors (Johnston et al. 2012; Yu et al. 2013). Proteins can be strongly adsorbed onto extremely hydrophobic surfaces through a mechanism known as the hydrophobic interaction. This process involves a favorable entropic contribution resulting from the release of water molecules that initially formed the hydration sphere around the protein (Nakanishi et al. 2001). However, mineral dusts do not typically exhibit such high degrees of hydrophobicity (Fubini and Areán, 1999). Hydrophilic surfaces promote protein adsorption and denaturation, as well as cell-surface adhesion, which could cause cellular injury (Salas et al. 2013). The plasma membrane of cells consists of a thin bilayer of amphiphilic phospholipids, proteins, and steroids, arranged such that the hydrophobic cores point inward, and the hydrophilic heads point outward towards the aqueous external environment and the internal cytoplasm (Ingólfsson et al. 2014). These hydrophilic head groups, which are typically negatively charged in vivo, may strongly interact with surface-exposed cations of mineral particles (typically replacing adsorbed water molecules) (Chen et al. 2023; Fubini & Areán, 1999). This interaction could potentially disrupt membrane structure and dynamics, leading to the breakdown or disruption of cellular membranes (Karlsson et al. 2013). Sahai (2002) proposed a thermodynamic approach to explain how various metal oxides interact with cell membranes. Their model predictions indicated that tetrahedral crystalline silica polymorphs exhibit membranolytic effects on liposomes, erythrocytes, macrophages, and lysosomes. In contrast, the octahedral silica polymorph (stishovite), amorphous silica, and oxides like TiO_2_, Al_2_O_3_, and Fe_2_O_3_ demonstrate lower cytotoxicity. The thermodynamics of interactions between oxide surfaces and cell membrane phospholipids (hydrophobic hydrocarbon chain attached to a hydrophilic phosphoryl polar head) relies on factors including the dielectric constant of the oxide, the Pauling bond strength-to-bond length ratio of the oxide, and the charge-to-radius ratio of the adsorbing ion. The last two parameters are influenced by the crystal structure and crystal chemistry (Schaufuss et al. 2000).

### Surface charge

Surface charge is a key factor affecting the reactivity of mineral dust in biological systems (Chung et al. 2007). Mechanical fracture of crystals can generate several reactive species on the surface (mechanochemical activation), which represent localized surface states that contribute to the surface charge (Delogu 2011). When a freshly cleaved surface is exposed to ambient conditions, highly reactive “dangling” bonds on the surface quickly bond with species from the surrounding medium (Schaufuss et al. 2000; Tessum et al. 2014). Considering a silicate surface in contact with water for simplicity, spectroscopic studies show that the first layer of water molecules on an oxide surface dissociate into H^+^ and OH^−^ ions, indicating the onset of surface charge (Henrich 1985). In the context of ionic solids, the presence of exposed, coordinatively unsaturated, cations and anions on their surface serve as electron acceptors and provide interaction points for electron donors (Brown et al. 1999; Stair 1982). Similarly, surface-exposed anions have the ability to interact with electron acceptors or dipolar molecules (Stair 1982). At the interface between solid particles and biological fluids, the ionic composition of the surface layer may be altered due to the preferential transfer of cations or anions into the aqueous liquid phase. This process disrupts the electroneutrality of the interface. This phenomenon results in the solid surface acquiring an electric charge (Ferraris et al. 2018; Stair 1982). The electrokinetic potential, often referred to as the zeta potential, can reach several hundred millivolts and plays a substantial role in influencing the interaction between mineral particles and living cells (Ferraris et al. 2018; Jiang et al. 2009). A high affinity for cellular membranes might increase the uptake of the particles by cells, leading to higher intracellular concentrations and potential toxicity (Chung et al. 2007). Light and Wei (Light and Wei 1977) discovered a strong positive relationship between the zeta potential of asbestos fibers and their ability to break down the membrane of red blood cells. We will explore the surface charge effect in more detail for the different components of the coal dust in subsequent sections.

### Adsorption capacity

Respirable dust particles can adsorb and concentrate endogenous (biological) molecules and metal ions on their surfaces, which may affect their pathogenicity (Mudgal et al. 2010). The level of adsorption depends on factors such as the specific surface area of particles, chemical composition, whether it is hydrophilic or hydrophobic, and its zeta potential (Fubini and Areán, 1999). This phenomenon can make the particles more toxic, as they can function as carriers, delivering other harmful substances deep into the respiratory system. One such metal that is known to accumulate on the surface of inhaled dusts is endogenous iron, which can cause radical damage to DNA and cell organelles through the Haber–Weiss cycle (Imlay et al. 1988; Toyokuni 1996). Ghio et al. (1990) proposed that this accumulation of iron could explain the higher rates of tuberculosis among silicate workers, as the iron complexed by the inhaled dust particles may be made available to dormant mycobacteria. Another example of potentially harmful substances that can adsorb on the airborne particulates are polycyclic aromatic hydrocarbons (PAHs), which can enhance ROS generation through metabolism (Abbas et al. 2018; Baulig et al. 2007; Boers et al. 2005). The elevated risk of lung cancer among individuals exposed to silica and asbestos dust, compared to non-smokers, has been linked to the adsorption of PAHs on these mineral particles (Abbas et al. 2018; Boers et al. 2005). Nitric oxide, a common atmospheric contaminant, can be absorbed by airborne particles such as mineral dusts (Robertson et al. 1982). When inhaled, the particulates with attached nitric oxide can disrupt the metabolic regulation of this essential biological molecule, potentially increasing the generation of harmful free radicals (Kameda et al. 2016).

### Solubility

The solubility of respirable dust in the respiratory system plays a crucial role in determining its toxicity. It affects how long particles stay in the lungs, their interaction with lung cells and tissues, and the subsequent biological responses (Giordani et al. 2022). Solubility of inhaled particles is strongly influenced by the complex composition of intracellular and extracellular fluids encountered by the particles, including pH, ionic strength and composition, and organic molecule contents (e.g., proteins, amino acids, antioxidants) (Giordani et al. 2022; Ortega et al. 2014). Soluble particles can dissolve in the lung fluid, releasing potentially toxic ions or compounds (Zhong et al. 2017). These ions or compounds can then enter the bloodstream and be distributed throughout the body, which can increase the range of potential toxic effects beyond the lungs (Giordani et al. 2022; Knaapen et al. 2002). As a case in point, the dissolution of the coal dust in the lung fluid can release iron, a widely recognized transition metal, which can be bioavailable for oxidant formation via Fenton, Haber–Weiss, or autoxidation processes (Huang 2003). Huang et al. (1998) characterized bioavailable iron (BAI) as the iron released in a 10 mM phosphate solution with a pH of 4.5, intended to simulate the conditions of phagolysosomes within cells. The human respiratory system has mechanisms, like the mucociliary escalator, to clear particles. Insoluble particles can evade these clearance mechanisms, leading to prolonged retention in the lungs (bio-persistence) (Oberdörster 1988). Over time, this can lead to chronic inflammation and other lung diseases (Moller et al. 2012; Ortega et al. 2014). It’s important to note that while surface layers or species of these particles may undergo partial dissolution, the core or primary mass of the particle often remains intact, contributing to the bio-persistence.

### Chemical composition

The primary chemical factors that determine the pathogenicity of particulates are linked to the interactions that occur at the interface between the mineral particle and lung tissue. Therefore, the surface chemical composition, which can notably differ from the underlying material or internal composition, and the presence of active surface states are the major chemical drivers of the biological response (Liu and Liu 2020). The elemental composition of respirable coal dust can differ depending on the geographical location of the coal deposit, proximity to other minerals and the rank of coal (Orem and Finkelman 2003; Sarver et al. 2021). This means that coal dust can contain various amounts of volatile organic compounds, PAHs, and other potentially harmful substances (Sarver et al. 2021; Zhang et al. 2015). Typically, the most prevalent elements in respirable particulates are Fe, Cu, Al, and Si-rich particles plus elemental carbon and organic matter (Roy et al. 2019; Song et al. 2016). The dominance of O, Si, and Al has been reported in flaky and irregular particles indicating the aluminosilicate nature of such particles (Pachauri et al. 2013).

A correlation between the presence of transition metals such as Co, Cu, Cr, Fe, Mn, Ni, V, Pb, As, and Zn in particulate matters and ROS production has been reported in previous works (Gosselin and Zagury 2020; Wilson et al. 2002). Such correlation has been attributed to variable oxidation states of these metals, which is a key factor to induce ROS generation (Rice-Evans et al. 1991; Szigeti et al. 2015). Mixed dusts often consist of various elements that can enhance each other’s effects when combined (synergistic effect). For example, fresh fractured quartz with trace levels of iron and the mix of metallic cobalt and tungsten carbide shows enhanced toxicity due to their combined interactions (Castranova et al. 1997; Fubini & Areán, 1999). Trechera et al. (2020) evaluated the contributions of the various elements in the oxidative potential of respirable dust samples and reported that Fe, Si, Mn, Ti, and Ba are associated with high potential impacts on human health. Their results showed that Fe is the element with the highest contribution to the oxidative stress induced by the respirable particles. Another concern is the possibility of DPM-dust attachment especially in the underground mines, which can complicate the surface chemistry and health impacts of the dust particles (Gaillard et al. 2019). DPM is a complex and variable mixture of elemental carbon, organic compounds (PAHs, Nitro-PAHs, aliphatic- and oxygenated hydrocarbons), sulfates, and other elements (Fe, Zn, Cu, Mg, Al, Cr, Na, Ca) (Viskup et al. 2020; Wang et al. 2019). It is also noteworthy that the surface state of a particle is influenced by its history, including how it was generated, any weathering or processing it has undergone, and any contaminants present. Therefore, particles with the same overall composition can have markedly different surface reactivity and levels of toxicity (Fubini and Fenoglio 2007).

### pH

Inhaled dust can influence the pH of the surrounding environment, i.e., biological fluids. An altered pH can affect lung physiology and can also lead to the leaching of toxic constituents from the dust particles. Depending on the mineral content of the dust (basic, e.g., calcite, dolomite or acidic, e.g., pyrite), the pH of the lung fluids may vary resulting in some variations in the dissolution and uptake of the dust particles (Hettiarachchi et al. 2018).

## Coal-specific issues

Coal is a variable heterogeneous mixture of organic carbon and various inorganic minerals (Tishmack and Burns 2004). The rank of coal is a measure of its maturity, and it increases from peat to lignite, sub-bituminous, bituminous, and anthracite (Cheng et al. 2017; Given 1984). As the rank of coal increases, there is a corresponding increase in the ratio of carbon to other chemicals and mineral contaminants. While coal primarily consists of carbon, coal mine dust contains hydrogen, oxygen, nitrogen, trace metals, and inorganic minerals, including quartz. Trace metals found in coal dust can include Cd, Sb, B, Al, Cu, Ni, Fe, Zn, and Pb, some of which can be cytotoxic and carcinogenic by acting as catalysts in Fenton-like reactions (Cohen 1985; Grayson 1991; Zhang et al. 2018). Coal contains various oxygen-containing functional groups like hydroxyl (–OH) with the infrared (IR) absorption band at 3400–3650 cm^− 1^, carbonyl (C = O, 1680–1710 cm^− 1^), and carboxylic (–COOH, 1710 cm^− 1^) groups (Given 1984; Levine et al. 1982; Orem and Finkelman 2003). In addition, coal contains aromatic nucleus (C = C) structures, aromatic C–H structures, and aliphatic chains (CH_3_, CH_2_) with IR peaks at 1610 cm^− 1^, 3046 cm^− 1^, and 2924 cm^− 1^, respectively (Given 1984; Xu et al. 2017). The IR peak of quartz (Si–O) in coal appears at 800 cm^− 1^ (Xu et al. 2017).

**Fig. 2:**
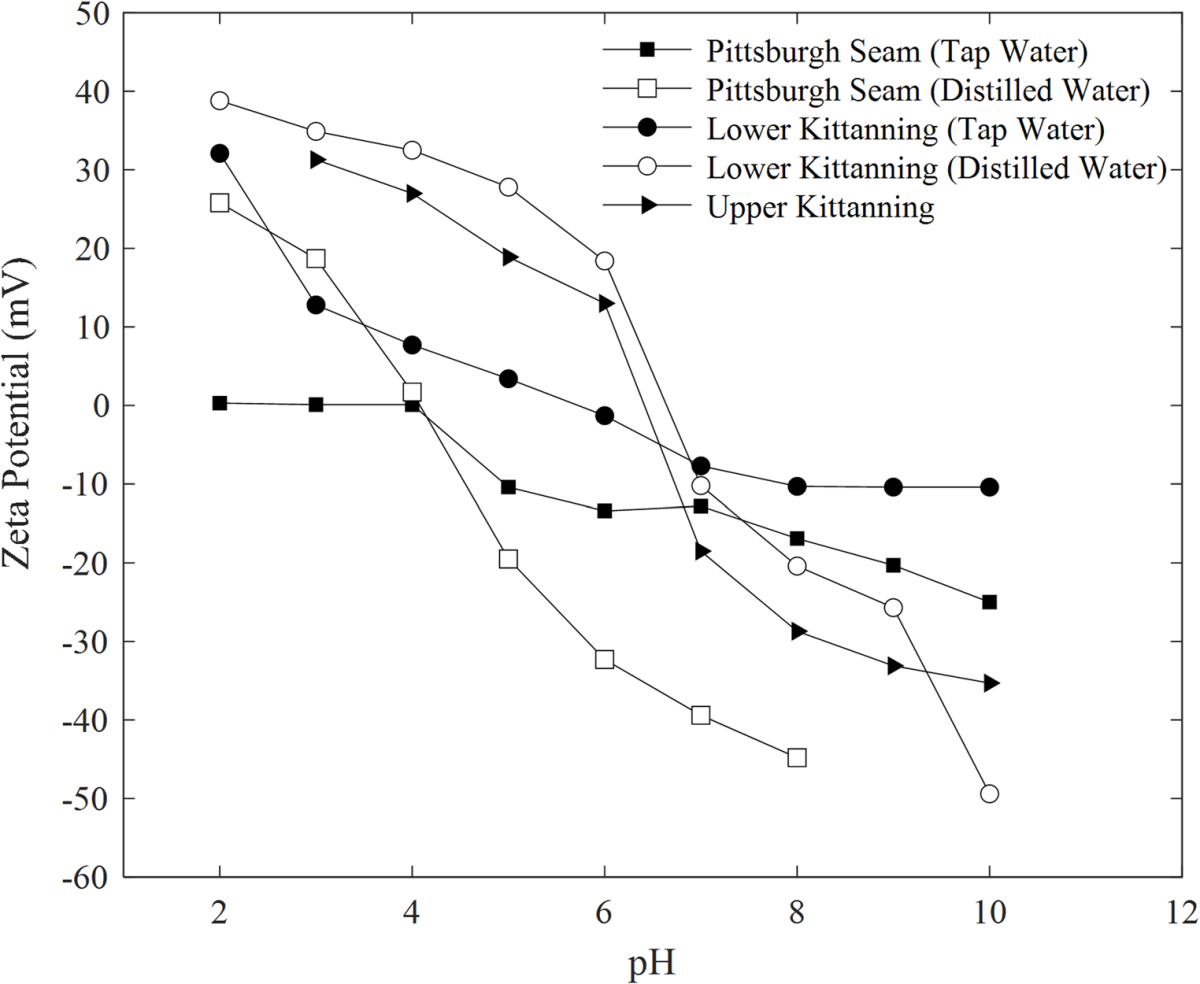
Effect of water quality on zeta potential of coal (reproduced from Arnold and Aplan 1986)

The oxidation of coal involves several processes, including the chemisorption of oxygen on the pore surfaces, the formation of carbon–oxygen compounds, the breakdown of unstable solid oxygenated intermediates into gaseous products and stable solid complexes, along with the formation of new active sites for oxidation (Beafore 1984; Dou and Zhong 2017). The rate of oxidation is further enhanced as the specific surface area of coal is increased through comminution (Wang et al. 2003). Coal shows a marked increase in hydrophilicity upon oxidation (Wei et al. 1992). The quantitative index of how oxidation increases the surface energy of the coal can be correlated with the initial carbon and oxygen content of the coal (Fuerstenau et al. 1988). Surface charge of coal changes with pH and water quality. A negative surface charge on coal emerges when acidic surface functional groups, e.g., carboxyl with a pK_a_ value of 4 dissociate (DeRuiter 2005), while the positive surface charge owes to the protonation of basic nitrogen-containing functional groups, such as pyridine (C₅H₅N) (Levine et al. 1982). Figure 2 presents the effect of water quality on zeta potential (representing surface charge) of coal (Arnold and Aplan 1986). Dalal et al. (1995) showed that five different coal mine dust samples differed markedly in their potential to generate •OH. A study by NIOSH showed that the prevalence of CWP is coal-rank dependent following high-rank coal > low-rank coal > medium- rank coal (Attfield et al. 2011). Anthracite coal mining has been associated with higher rates of pneumoconiosis than those found in bituminous coal miners (Doney et al. 2019). Bituminous mines, which tend to have a higher clay content compared to anthracite mines, often contain quartz particles. When quartz is cut along with coal in these deposits, the quartz particles are more likely to be coated with clays, which can render them less biologically active (Wallace et al. 1994). Another contributing factor would be the content of surface free radicals, which was reported as being higher for anthracite coal particles compared to lower-ranked coals (McCunney et al. 2009; Ross and Murray 2004).

The reason CWP is highly rank dependent, and the correlation between coal rank and cell cytotoxicity have not been studied yet. Epidemiological studies have confirmed that the rank of coal influences the rates of CWP among coal workers, suggesting the carbon content of coal as a critical factor in studying CWP risk (McCunney et al. 2009). This was disputed by Joy et al. (Joy et al. 2012) who found a lower incidence of CWP in Australia despite their higher coal rank compared with those in the U.S. Subsequent studies in the U.S. and Australia found quartz to be an alternative influential factor (Blackley et al. 2014; Graber et al. 2017). The risk of CWP has also been linked to the BAI content of coal, which as noted before can catalyze the production of ROS (McCunney et al. 2009). The presence of carbon-centered free radicals in coal has been documented in several studies (Dalal et al. 1995; Duan et al. 2023; Xi et al. 2022). These studies suggest that the concentration of free radicals increases with the mechanical breaking of chemical bonds, so that freshly generated coal particles are more toxic. However, the coal-based carbon-centered radicals are regarded as stable and confined in the coal and may be biologically neutral (Green et al. 2012; Tian et al. 2009).

Sulfur (S) is another prominent harmful element in coal, leading to significant environmental pollution through the emission of SO_2_ during combustion (Zhang et al. 2016). Sulfur in coal exists as inorganic and organic types. If liberated, inorganic sulfur, found mainly as pyrite, can be removed through conventional coal washing, while the removal of organic sulfur, which exists either as small molecule compounds or as part of a complex macromolecular structure forming C-S bonds, is more challenging due to its intricate chemical composition (Calkins 1994). The main forms of organic sulfur in coal include mercaptans, disulphides, thioethers, sulfoxides, sulfones, thiophenes, and sulfonates, among others (Zhang et al. 2016). Cohn et al. (2006b) observed that the release of Fe^2+^ into solution can be correlated with the sulfur content of coal and coals containing less than 1% sulfur do not form detectible H_2_O_2_ or •OH, while coals that contain more than 1% sulfur form •OH and H_2_O_2_ and degrade ribonucleic acid (RNA). They reported that coal samples that do not contain iron do not induce the generation of ROS nor degrade RNA (Cohn et al. 2006b), which may indicate that the coal-induced production of •OH and H_2_O_2_ involves iron (pyrite) and not the organic fraction of coal.

Organic aromatic compounds including benzene, methylene, phenol, and phenanthrene found in the coal atmosphere have the potential to be adsorbed onto the surface of respirable coal particles, potentially influencing their biological activity (Castranova and Vallyathan 2000). In addition, coal is a major source of environmentally persistent free radicals (EPFRs), organic free radicals stabilized on or inside particles (Filippi et al. 2022). The formation of EPFRs in coal is a complex process that involves the breakdown of organic matter during combustion. The formation of EPFRs is believed to occur through the interaction of oxygen with organic molecules in coal. These EPFRs can remain stable for several years, due to their stabilization on coal particle surfaces, which also facilitates their potential release into the environment during coal mining, transportation, and combustion (Pan et al. 2019). EPFRs can have harmful effects on human health including reaction with biological molecules, such as DNA, proteins, and lipids, causing damage that can lead to cell death (Odinga et al. 2020). They have also been shown to contribute to the formation of ROS (Khachatryan et al. 2011). The exact mechanisms of EPFR toxicity and environmental behavior are still being studied, and more research is needed to fully understand their impact (Vejerano et al. 2018).

## Pyrite-specific issues

Pyrite (iron sulfide, FeS_2_) is a common inorganic component in coal with variable distribution in different coal dust samples depending on the geographical location (Vassilev and Vassileva 1996). It has been reported that pyrite surface oxidation significantly affects its surface charge development (Bebie et al. 1998; Fornasiero et al. 1992). Fornasiero et al. (1992) reported that when exposed to oxygen, as a result of gradual oxidation, pyrite surface develops a surface charge like that of iron oxyhydroxide (FeOOH) with the isoelectric point (IEP) of 7.8. They found that through oxidative dissolution, the pyrite surface acquires an FeOOH coating, which covers the surface to the point where the IEP of pyrite shifts from pH 1.2 ± 0.4 for “non-oxidized” surface to pH 7 for “oxidized” surface. The oxidative dissolution of pyrite can proceed through two potential reaction paths: one involving molecular oxygen as the oxidant, as described in Rxn. (2), and another where dissolved ferric iron serves as the oxidant, as detailed in Rxn. (3). Notably, this ferric iron can be generated through the oxidation of ferrous iron following Rxn. (4) (Harrington et al. 2012).

2 $$\:2\mathrm{Fe}\mathrm{S}_{2}+7\mathrm{O}_{2}+2\mathrm{H}_{2}\mathrm{O}\to\:2\mathrm{Fe}^{2+}+4\mathrm{S}\mathrm{O}_{4}^{2-}+4\mathrm{H}^{+}$$

3 $$\:\mathrm{Fe}\mathrm{S}_{2}+14\mathrm{Fe}^{3+}+8\mathrm{H}_{2}\mathrm{O}\to\:15\mathrm{Fe}^{2+}+2\mathrm{S}\mathrm{O}_{4}^{2-}+16\mathrm{H}^{+}$$

4 $$\:2\mathrm{Fe}^{2+}+\frac{1}{2}\mathrm{O}_{2}+2\mathrm{H}^{+}\to\:2\mathrm{Fe}^{3+}+\mathrm{H}_{2}\mathrm{O}$$

The oxidative dissolution of pyrite releases ferrous iron into the solution, which then interacts with dissolved molecular oxygen leading to the Haber–Weiss reaction mechanism (Kehrer 2000) and eventually the formation of H_2_O_2_ (Rxns. 5 and 6), intermediated by superoxide ($\:{{\mathrm{O}}_{2}^{{\bullet\:}}}^{-}$). The other pathway for the formation of H_2_O_2_, which follows the same reactions, is through the reduction of oxygen by surface-bound Fe^2+^ (structural or sorbed) (Cohn et al. 2006a). Surface-complexed Fe²⁺ is more reductive than dissolved Fe²⁺, primarily due to the donation of electron density from the ligand to the Fe²⁺. This enlarges the energy of the electron-donating Fe *3d* orbitals, enhancing the capacity for electron transfer (Huang et al. 2021). It has been reported that pyrite can also induce the generation of H_2_O_2_ in the absence of molecular oxygen (Borda et al. 2003). The proposed mechanism is driven by a reaction between water and a sulfur-deficient defect (Guevremont et al. 1997) on the pyrite surface (Rxns. 7 and 8) (Borda et al. 2003). Normally, each sulfur atom in the disulfide moiety of pyrite has an oxidation state of −1, while sulfur associated with a sulfur-deficient defect has an oxidation state of −2, which requires some iron on the pyrite surface to be in the + 3 state, i.e., as Fe^3+^ (Borda et al. 2003; Cohn et al. 2006a).

5 $$\:{\mathrm{F}\mathrm{e}}^{2+} {\mathrm{(aq/pyrite)}}+ {\mathrm{O}}_{2}\:\to\:\:{\mathrm{F}\mathrm{e}}^{3+} {\mathrm{(aq/pyrite)}} +\:\:{\mathrm{O}_2^{\bullet}} ^{-}$$

6 $$\:{\mathrm{F}\mathrm{e}}^{2+} {\mathrm{(aq/pyrite)}}+ {\mathrm{O}_2^{\bullet}} ^{-} + 2 \,{\mathrm{H}}^{+}\:\to\:\:{\mathrm{F}\mathrm{e}}^{3+} {\mathrm{(aq/pyrite)}} +\:\: {\mathrm{H}_{2}}{\mathrm{O}_{2}}$$

7 $$\:{\mathrm{F}\mathrm{e}}^{3+} {\mathrm{(pyrite)}}+ {\mathrm{H}_{2}{\mathrm{O}}}\:\to\:\:{\mathrm{F}\mathrm{e}}^{2+} {\mathrm{(pyrite)}} +\:\: \bullet {\mathrm{OH}} + {\mathrm{H}}^{+}$$

8 $$2\bullet{\mathrm{OH}} \to\:\: {\mathrm{H}_{2}}{\mathrm{O}_{2}}$$

**Fig. 3:**
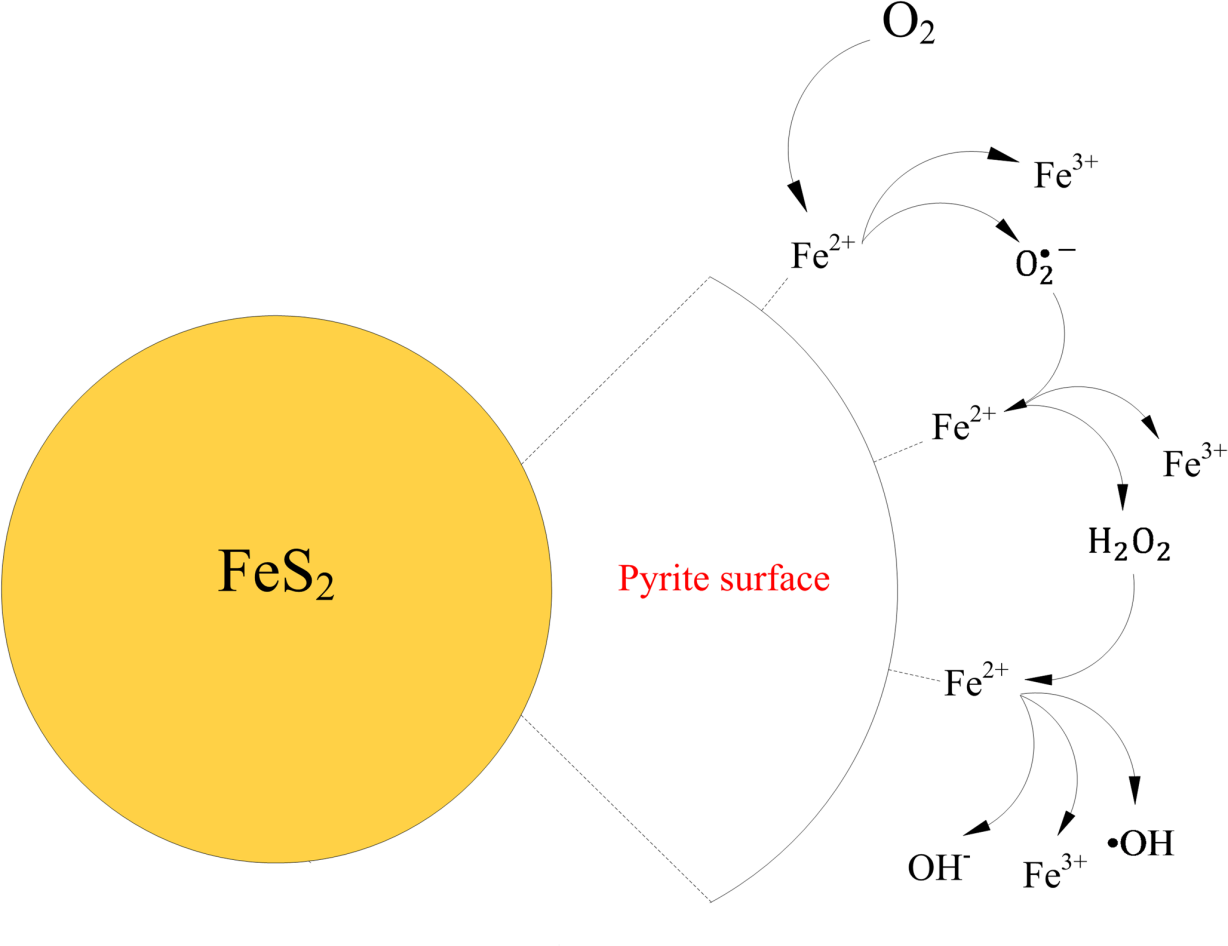
Pyrite surface oxidation and the generation of •OH

Hydrogen peroxide allows for the formation of •OH via the Fenton reaction (Rxn. 9) (Cohen 1985). Iron-catalyzed generation of •OH by the Haber–Weiss cycle needs iron only in trace amounts, and the produced •OH can overload the antioxidant defense mechanisms of living cells (Wardman and Candeias 1996). Figure 3 shows the steps for oxygen-induced pyrite surface oxidation and the generation of •OH following the Fenton reaction. The fate of H_2_O_2_ is dependent on the relative availability of ferrous and ferric iron; so that while its reaction with Fe^2+^ results in the generation of •OH, its reaction with Fe^3+^ facilitates its breakdown into water and oxygen (Rxn. 10) (Harrington et al. 2012; Schoonen et al. 2010).

9 $$\:{\mathrm{F}\mathrm{e}}^{2+} + {\mathrm{H}_{2}}{\mathrm{O}_{2}} \:\to\:\:\bullet {\mathrm{OH}} +{\mathrm{OH}}^{-}\:\:{\mathrm{F}\mathrm{e}}^{3+}$$

10 $$2{\mathrm{F}\mathrm{e}}^{3+} +3{\mathrm{H}_{2}}{\mathrm{O}_{2}} \:\to\:\:2{\mathrm{F}\mathrm{e}}^{2+} + 2{\mathrm{H}_2}{\mathrm{O}} +2{\mathrm{O}_2}+ 2{\mathrm{H}}^{+}$$

Hydroxyl radical formation from pyrite oxidation can also be strongly affected by solution pH (Bonnissel-Gissinger et al. 1998; Schoonen et al. 2000). Under acidic conditions (pH ≤ 4), the solubility of pyrite increases, enhancing the availability of Fe²⁺ (and SO_4_^2−^) ions and potentially elevating the generation of •OH (Bonnissel-Gissinger et al. 1998). Moreover, H_2_O_2_ maintains stability in an acidic environment, preserving its role in fostering •OH formation (Borda et al. 2001). The acidic condition also facilitates the cyclical oxidation and reduction of Fe³⁺/Fe²⁺ (Stumm and Morgan 2012), promoting a continuous catalytic cycle. Conversely, alkaline conditions may hinder the continuity of this catalytic cycle by obstructing the reduction of Fe³⁺ back to Fe²⁺ (Stumm and Morgan 2012), formation of ferric hydroxide, and fostering faster decomposition of H_2_O_2_ (Galbács and Csányi 1983). Pyrite •OH production can also be promoted in an illuminated environment due to the enhanced generation of O_2_^•−^ radicals by photoelectrons (Liu et al. 2024).

Dusts containing pyrite and siderite have shown a greater potential to induce more severe cell dysfunction compared to dusts containing Fe^2+^–OH, Fe^2+^–sulfate, and Fe^2+/3+^–(phyllo)silicate. This effect may be attributable to the presence of more reactive structural Fe^2+^ (Sun et al. 2022). In biological systems, high concentrations of iron can lead to oxidation of biomolecules (Cohn et al. 2006b). Furthermore, iron found in association with substances such as quartz (McCunney et al. 2009), iron oxides (Schins 2002), and iron sulfides (Cohn et al. 2006b) has been identified as a key reactant in the mechanisms that cause lung injury. This raises the question about the potential of similar reactions occurring in coal that contains pyrite. Cohn et al. (2006b) investigated the role of mineral pyrite in various amounts in the formation of ROS in coal samples. They found that the iron content of coal correlates with the association of pyrite, and coal samples that contain a higher content of iron induce a higher rate of ROS generation, which is not scavenged by the organic fraction of the coals. Harrington et al. (Harrington et al. 2012) have indicated the formation of •OH on pyrite surfaces in simulated lung fluid (SLF) and water. They found that •OH are also formed through pyrite oxidation in SLF but at lower concentrations than in water. Unfortunately, these researchers used mineral pyrite in their experiments instead of coal-pyrite. Esposito (1986) demonstrated differences in dissolution behavior and surface morphology between coal-pyrites and mineral pyrite. This suggests that some coal-pyrites may generate •OH more aggressively than mineral pyrite. Sokolović et al. (2006, 2012) reported the IEP of coal-pyrite at pH about 8.2. They attributed the elevated IEP value measured for coal-pyrite to the abundance of Fe^2+^ ions, a consequence of the high solubility of coal-pyrite. Such findings are consistent with the results of Jiang et al. (Jiang et al. 1998).

**Fig. 4:**
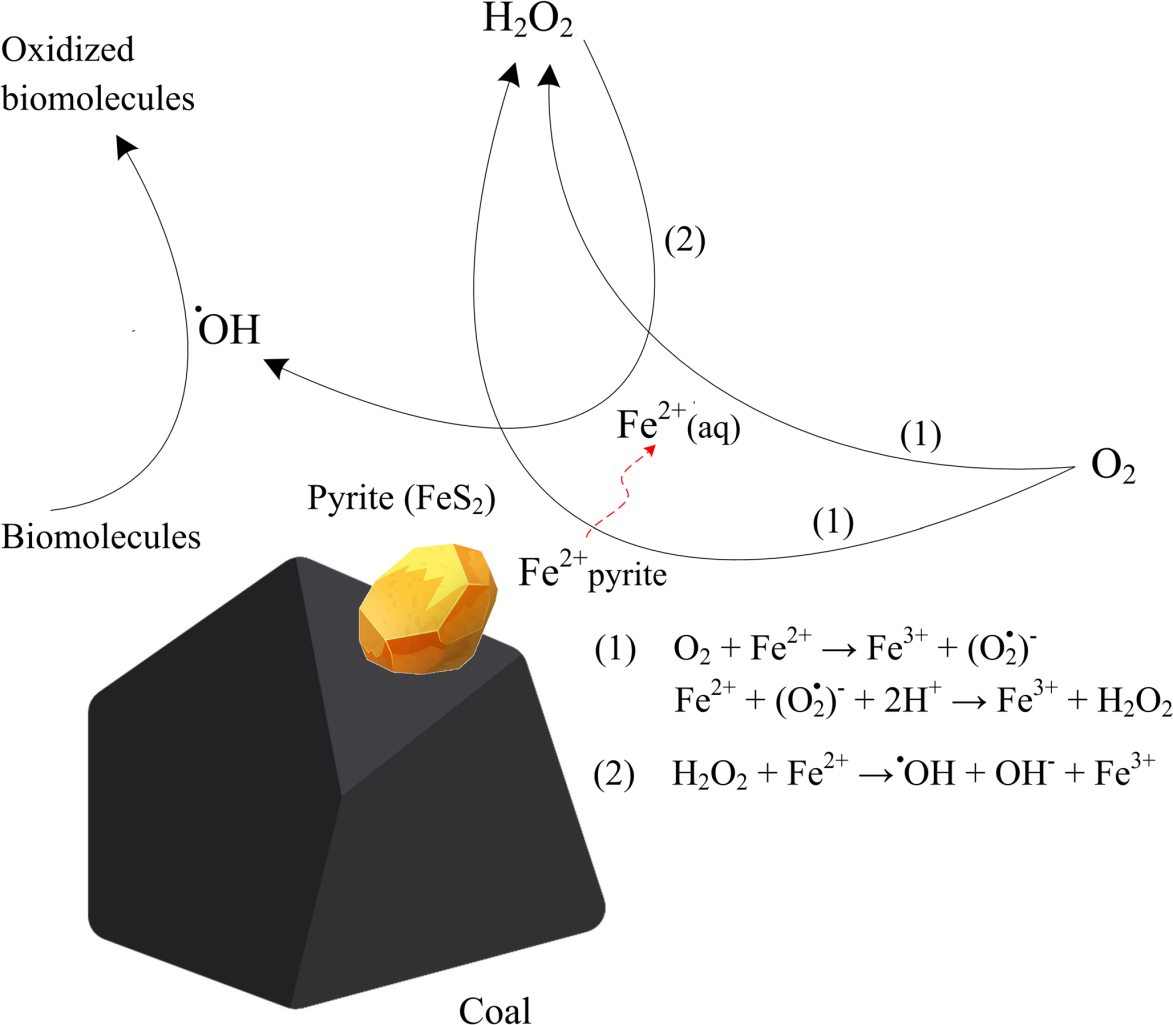
Mechanisms through which coal-pyrite reacts with dissolved oxygen to produce H_2_O_2_ and •OH, where •OH is responsible for the degradation of biomolecules (reproduced from Cohn et al. 2006b)

The prevalence of CWP among various coalmines has shown to positively correlate with average levels of BAI and the amount of pyrite in the coals (Huang et al. 2005; Morgan 1968; Zhang and Huang 2003). This implies that coal dust’s harmful effects can be partially attributed to pyrite. However, coal dusts containing iron in the form of iron-sulfate have exhibited reduced toxicity towards lung epithelial cells. This has been attributed to either limited iron availability or increased solubility (Sun et al. 2022). Figure 4 summarizes the mechanisms through which coal-pyrite reacts with dissolved oxygen to produce H_2_O_2_ and •OH following the Haber–Weiss cycle and Fenton reaction (reproduced from Cohn et al. 2006b). The presence of other metals like arsenic, potentially related to the pyrite content, might also contribute to ROS generation in coal samples (Cohn et al. 2006b; Finkelman 1995; Martin et al. 2014).

## Quartz-specific issues

Quartz (SiO_2_) is a major mineral component in coal dust (Trechera et al. 2020; Zazouli et al. 2021). Alpha(α)-quartz is the most common and stable form of crystalline silica with a trigonal crystal system (Le Page and Donnay 1976; Palyi et al. 2004). It is well established that intensive exposure to quartz dust can lead to respiratory and autoimmune diseases including silicosis, rheumatoid arthritis, and lupus (Fubini 1998; Fubini and Areán 1999; Maeda et al. 2010; Pavan et al. 2019). According to a report by the Guardian (The Guardian 2020), about 100 workers die every year in the U.S. due to silicosis. Quartz dust can also promote lung cancer (Koskela et al. 1994; Poinen-Rughooputh et al. 2016). The International Agency for Research on Cancer (IARC) (Wilbourn et al. 1997) has classified quartz and cristobalite (a polymorph of crystalline silica) as Group 1 carcinogens, meaning that there is sufficient evidence that they cause cancer in humans. Experimental in vitro studies have shown that quartz dust exposure can damage cell membranes and DNA in alveolar macrophages (Ziemann et al. 2017), form ROS in lung epithelial cells, and release inflammatory cytokines (Schins et al. 2002). According to IARC (Wilbourn et al. 1997), the carcinogenicity and biological activity of crystalline silica may depend on the type of silica or on other factors including crystal defects, chemical functionalities, and association with other substances.

It is still unclear what makes a specific quartz source pathogenic (Jennings and Flahive 2004). In general, crystallinity and various surface features, including form and micromorphology (fractured surfaces, sharp edges, poorly coordinated ions, and surface defects), govern the surface reactivity and, therefore, the toxicity of mineral particles (Jennings and Flahive 2004; Turci et al. 2016). Turci et al. (2016) found that the biological activity of quartz dust is not solely due to crystallinity but also because of crystal fragmentation, specifically when conchoidal fractures are generated.

**Fig. 5:**
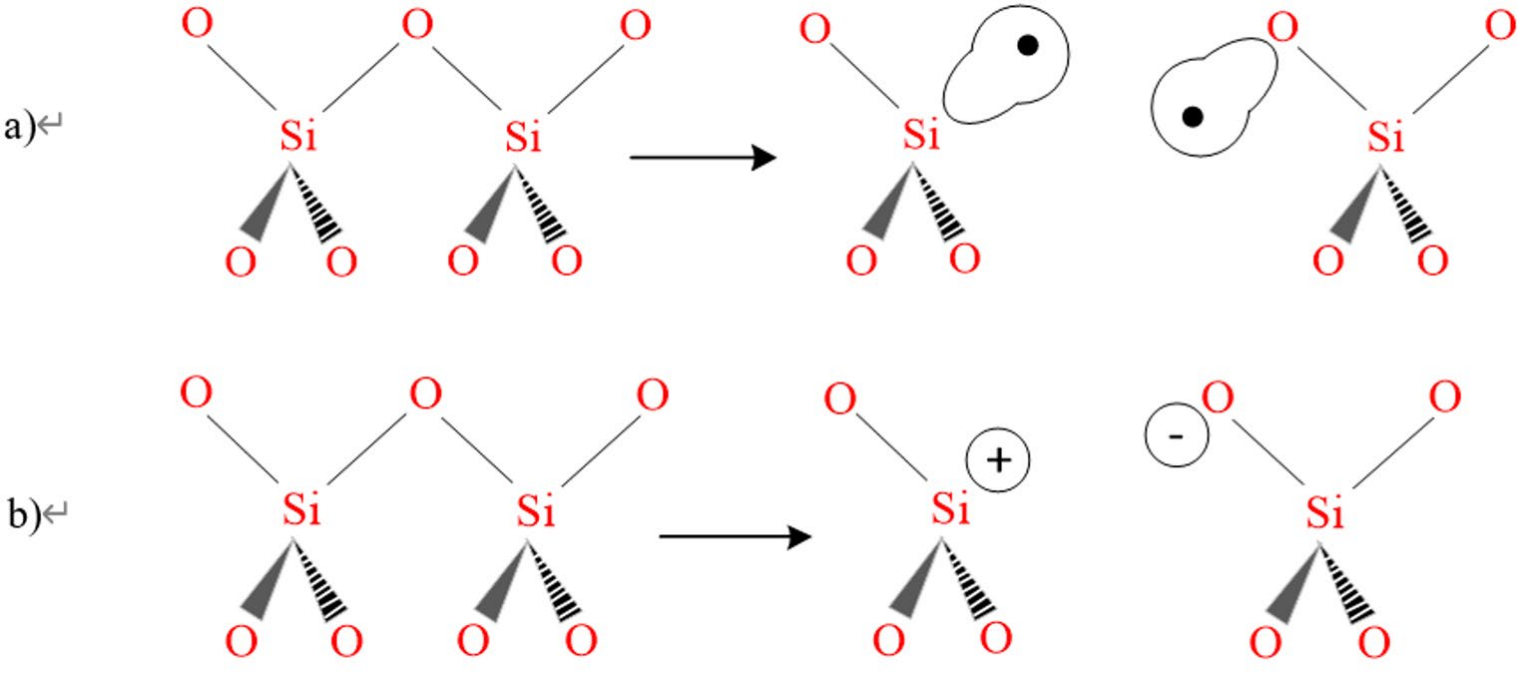
**a** Homolytic, **b** and heterolytic breakage of Si–O–Si bonds in quartz (reproduced from Fubini & Arean 1999)

**Fig. 6:**
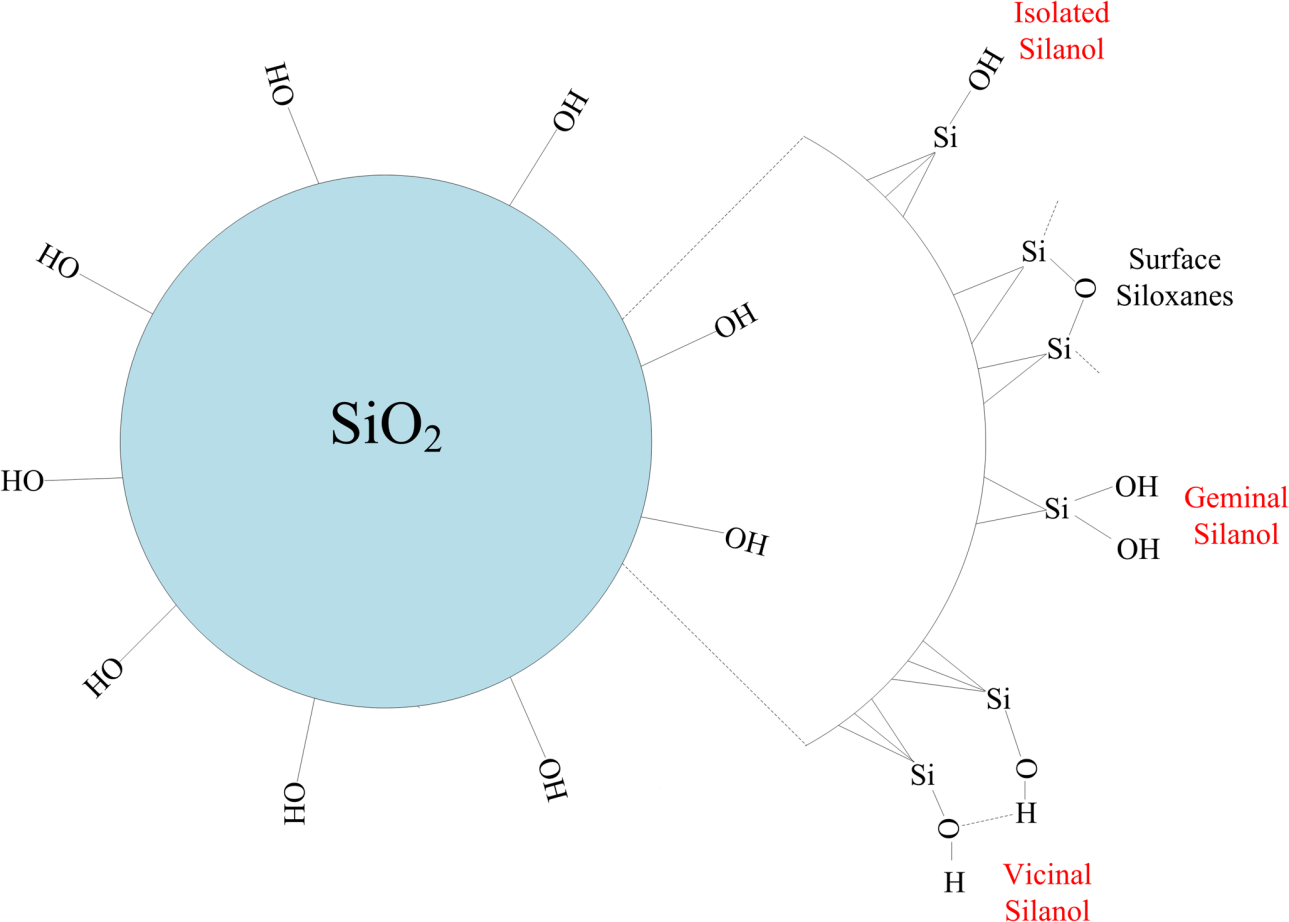
Quartz surface oxidation and the generation of silanol groups

**Fig. 7:**
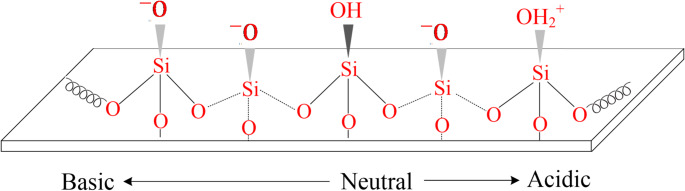
A schematic representation of quartz surface protonation at different pH conditions

Respirable quartz dust is typically generated when quartz-containing materials such as sandstone and granite are cut, ground, drilled, or crushed, causing large crystals to fracture irregularly with sharp edges and spikes (Jennings and Flahive 2004). The mechanical fracture of quartz crystals results in the breaking of Si–O–Si bonds, which connect the SiO_4_ tetrahedra. As a result, two distinct types of cleavages can occur: homolytic and heterolytic, as depicted in Fig. 5. Homolytic cleavage produces Si• and SiO• radicals, often called dangling bonds, while heterolytic cleavage generates electrically charged surface species, specifically Si^+^ and SiO^–^ (Fubini and Areán, 1999). These radicals on the freshly fractured quartz surface tend to form silanol groups (Si–OH) in the presence of water molecules, as represented in Fig. 6 (Castranova and Vallyathan 2000). Such dangling bonds and/or charged surface species on the freshly cleaved quartz surface exhibit a peculiar reactivity (Fubini et al. 1990), which has been linked to acute silicosis (Vallyathan et al. 1995; Fubini and Areán 1999). In addition, the charge separation occurring on the fractured quartz surface enhances its hydrophilicity, which influences its interaction with biological molecules (Fubini and Areán 1999). The silanol groups, due to their Brønsted acidity, play a crucial role in quartz surface charge development by either accepting or donating protons (H^+^ ions) depending on the pH of the surrounding medium, as depicted in Fig. 7 (Nolan et al. 1981). At low pH, silanol groups tend to accept protons, leaving the quartz surface with a positive charge (SiOH_2_^+^). At alkaline pH, silanol groups donate protons, resulting in a negatively charged surface (SiO^–^) (Michael and Williams 1984). The IEP for quartz is typically found around pH 2 (Yukselen-Aksoy and Kaya 2011), indicating that quartz surfaces are usually negatively charged in biological environments (pH 7.4), playing a significant role in disease generation (Nolan et al. 1981; Tsyganenko et al. 2000).

The exact mechanism of •OH production on the quartz surface is not fully understood, but there are proposed mechanisms. On the (001) surface of freshly fractured quartz, the formation of silicon-based radicals, e.g., SiO• radicals are believed to cause the generation of Si–OH, •OH and H_2_O_2_ in the presence of water molecules (Rxns. 11 to 13) (Hair 1975; Kalbanev et al. 1980; Narayanasamy and Kubicki 2005). However, studies involving electron paramagnetic resonance (ESR) spectroscopy have shown that SiO• radicals on the fresh quartz surface decay as function of time on exposure to the atmosphere, with a half-life of about 36 h (Dalai et al. 1986; Vallyathan 1997). The presence of water (moisture) may accelerate this decay process (Jennings and Flahive 2004). In experimental in vivo studies, freshly ground silicas have shown a higher degree of toxicity than aged ones (Castranova et al. 1996a, b; Vallyathan et al. 1995). The fracturing procedure and the components of the environment in which the fracturing takes place also influence the surface reactivity of quartz particles. A dry oxygen environment promotes the formation of surface radicals essential for the subsequent ROS generation, while a wet environment facilitates complete surface hydration at the site of broken bonds, with almost no formation of surface reactive species (Fubini 1998; Jennings and Flahive 2004). Mechanical fracturing is also believed to disrupt the arrangement of non-radical moieties such as siloxanes on the surface of quartz, which leads to disruption of cell membranes and cellular toxicity (Turci et al. 2016).

11 $$\:\equiv\:\mathrm{S}\mathrm{i}\mathrm{O}\bullet\:+\:{\mathrm{H}}_{2}\mathrm{O}\to\:\:\equiv\:\mathrm{S}\mathrm{i}\mathrm{O}\mathrm{H}+\:\bullet\:\mathrm{O}\mathrm{H}$$

12 $$\:\equiv\:\mathrm{S}\mathrm{i}\mathrm{O}\bullet\:\:+\:\bullet\:\mathrm{O}\mathrm{H}\to\:\:\equiv\:\mathrm{S}\mathrm{i}\mathrm{O}\mathrm{O}\mathrm{H}$$

13 $$\:\equiv\:\mathrm{S}\mathrm{i}\mathrm{O}\mathrm{O}\mathrm{H}+\:{\mathrm{H}}_{2}\mathrm{O}\to\:\:\equiv\:\mathrm{S}\mathrm{i}\mathrm{O}\bullet\:\:+\:{\mathrm{H}}_{2}{\mathrm{O}}_{2}$$

Several other physical and chemical properties of quartz contribute to its ability to cause disease, including hydrophilicity and the presence of metal impurities (Fubini and Fenoglio 2007). One notable example is the contamination of the coal-quartz with trace levels of iron, which often occurs due to the natural association of quartz with iron sulfides such as pyrite in coal deposits (Dai et al. 2021; Orem and Finkelman 2003). This trace iron contamination can enhance the quartz generation of •OH through a Fenton-like reaction, according to Rxn. (14) (Castranova et al. 1997). However, studies involving metal chelators have indicated that the quartz-induced formation of •OH stems from the direct reactivity of the fresh mineral surface, rather than the Fenton-like reaction (Shi et al. 1988). The surface reactivity and the biological activity of quartz of various origin are extremely variable, making it a “variable entity” with regards to its pathogenic activity in biological systems (Donaldson and Borm 1998; Fubini 1998; Jennings and Flahive 2004; Warheit et al. 2007).

14 $$\:{\mathrm{F}\mathrm{e}}^{2+}+{\mathrm{H}}_{2}{\mathrm{O}}_{2}\:\to\:\:{\mathrm{F}\mathrm{e}}^{3+}+\:\bullet\:\mathrm{O}\mathrm{H}+\:{\mathrm{O}\mathrm{H}}^{-}$$

Despite extensive toxicological investigations of quartz, few studies have used quartz that reflects the types found in industries with occupational quartz exposure. Much of the exposure involves mixed dusts, such as coal dust, where the quartz surface might be altered by the presence of substances like coal, clays, iron, or aluminum (Donaldson and Borm 1998).

## Clay-specific issues

Clays associated with coal may appear in various forms, such as kaolinite, chlorite, illite, montmorillonite, or mixed clay (Daniels and Altaner 1990; Gluskoter 1967; Sarver et al. 2021; Tishmack and Burns 2004; Vassilev and Vassileva 1996). Characterizing these clays is challenging due to their complex chemical and morphological characteristics (Zygarlicke and Steadman 1990). Naturally occurring in small sizes, clays can readily become respirable during mechanical activities like cutting, drilling, and grinding. Due to their low density, fine clay particles can remain airborne for extended periods, increasing the likelihood of inhalation. Clay particles hosting the transition metal ions like iron can generate •OH under certain conditions (Song et al. 2006; Zeng et al. 2017). The main mechanism involves the oxidation of structural iron (Fe^2+^) through the electron transfer to O_2_ (oxygenation) and the subsequent production of •OH following the Fenton-like reaction (Yuan et al. 2018; Zhao et al. 2021). Finer-sized clays produce more •OH due to their larger surface area, which facilitates more interaction with O_2_ (Gournis et al. 2002). Iron-rich montmorillonite has been observed to generate •OH in a suspension illuminated by near UV and visible light (Wang et al. 2013; Wu et al. 2008; Yu et al. 2021). The photolysis of $\:{\mathrm{Fe}{\left(\mathrm{OH}\right)}^{2+}}_{aq}$ complexes (that is the hydration product of Fe^3+^ ions extracted from the clay into solution) in the solution has been suggested to be the most probable mechanism for the formation of •OH from an iron-rich montmorillonite (Zhang et al. 2008). Recent studies have also documented the dark formation of •OH from iron-rich clays, which is attributed to the oxidation of structural Fe^2+^ (Yu et al. 2021; Yuan et al. 2018; Zeng et al. 2017). Clays hosting the transition metals can also produce EPFRs on the surface upon sorption and then transformation of the aromatic molecules including phenols and PAHs (Jia et al. 2016; Nwosu et al. 2016).

Prolonged exposure to high concentrations of certain types of clay can lead to the development of respiratory diseases like kaolinosis, a type of pneumoconiosis caused by the inhalation of kaolin dust (Lynch and McIver 1954; Sheers 1964). Considering that kaolin is chemically inert in its pure form, impurities within the kaolin, such as free silica, can contribute to its pathogenicity. However, despite a large number of radiographic and microscopic studies devoted to kaolinosis (Davies et al. 1984; Wells et al. 1985), research in this area has not led to a consensus. Some studies show that kaolin dust without crystalline silica has limited or significantly reduced pathogenic effects on the lungs (Altekruse et al. 1984; Sepulveda et al. 1983; Warraki and Herant 1963), while others still indicate a risk (Hale et al. 1956; Lesser et al. 1978). There is also evidence suggesting that aluminosilicate surface treatment of respirable quartz particles leads to remarkable reduction in their pathogenicity (Wallace et al. 1994). Based on an experimental in vivo study, Stone et al. (Stone et al. 2004) reported that pretreatment of quartz particles with extracts of either kaolin or coal mine dust remarkably reduces quartz-induced inflammatory responses. They suggested that the soluble elements in kaolin and coal mine dust, possibly cations, could mask the reactivity of the quartz surface.

## Opportunities for reducing •OH generation of coal dust

**Fig. 8:**
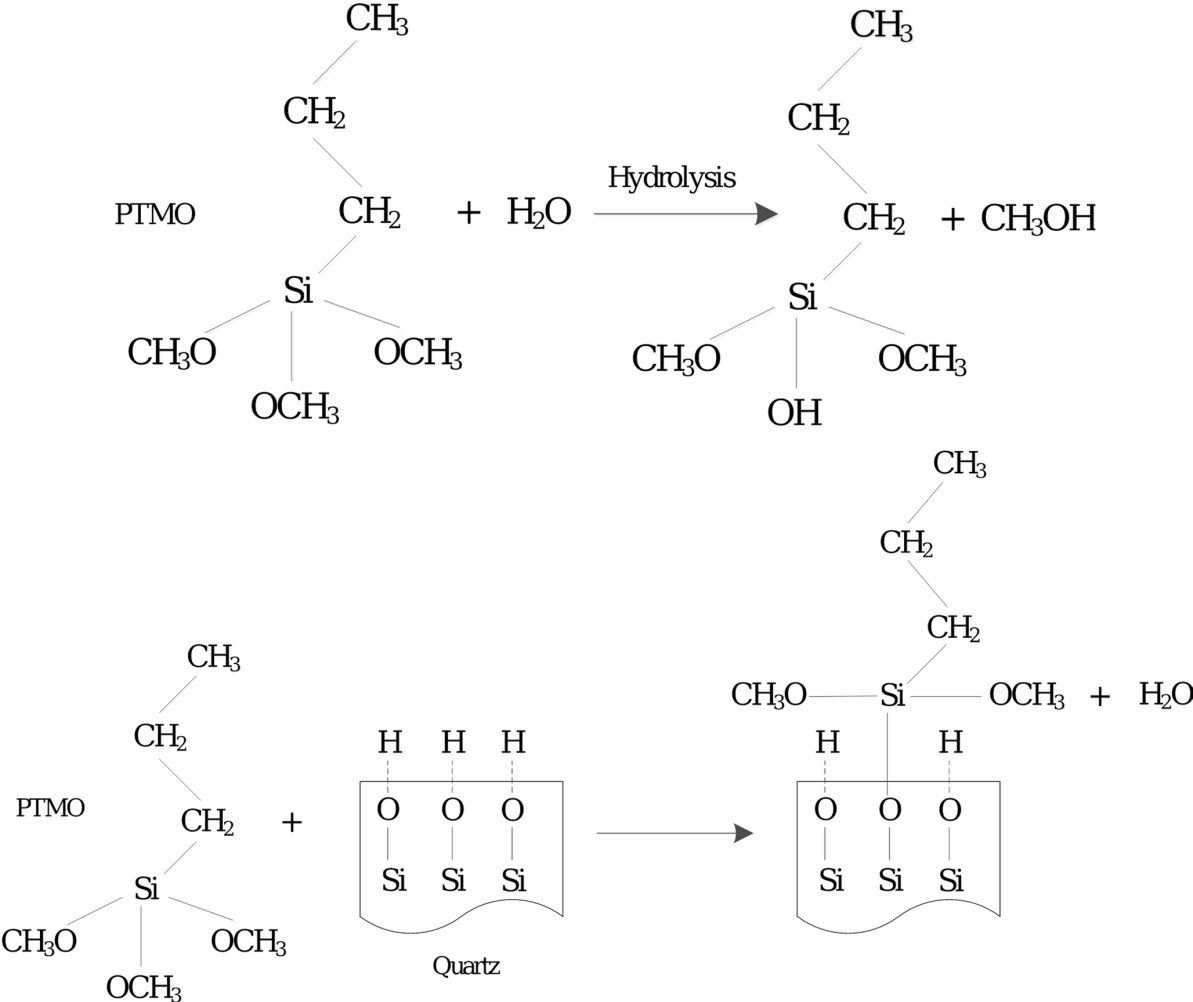
Hydrolysis of PTMO and its coating reaction with reactive silanol groups on the quartz surface (reproduced from Ziemann et al. 2017)

Reducing or inhibiting the coal dust generation of •OH radicals in mining presents opportunities to prevent the occurrence of coal mine related diseases (Liu and Liu 2020). Given that the generation of •OH in dust is linked to the surface oxidation of dust particulates, it is hypothesized that inhibiting or reducing the surface oxidation of coal dust components would lead to a corresponding reduction or elimination of •OH generation. The development and application of novel materials or coatings that neutralize or inhibit the formation of •OH radicals in coal dust could also be a promising approach. These materials could be added to the mix of spray water (dust suppression systems) as additives to effectively interact with the dust components in air, thereby altering their surface properties and reducing their capability to generate harmful radicals (Tessum and Raynor 2017). In this context, several mechanisms including the metal-micelle coating, blockage of surface-active sites, chelation of the surface metal ions, surface silanization, masking of surface-active sites with rare earth elements, and change in surface functional groups via polymer-coating have been proposed (Gulumian 2005). For further information, researchers are encouraged to read an extensive review by Gulumian (2005). For example, quartz •OH generation is linked to density and abundance of surface silanol groups/radicals. Data indicate that coatings including poly-2-vinylpyridine-N-oxide, aluminum lactate, Dynasylan^®^ PTMO (poly tetramethylene oxide), Dynasylan^®^ SIVO 160, and Prosil-28 that abolish these surface radicals are effective in reducing cytotoxicity and the ability of silica to activate pulmonary phagocytes (Nolan et al. 1981; Vallyathan et al. 1991; Ziemann et al. 2017). Figure 8 shows the hydrolysis of PTMO and its coating reaction with reactive silanol groups on the quartz surface. Quartz surfaces interacting with strong Lewis acids like AlCl_3_ or FeCl_3_, which react with $\:{\mathrm{S}\mathrm{i}\mathrm{O}}^{-}$ sites, also experience a decrease in biological activity (Nolan et al. 1981).

Jin et al. (2019) tested a new coal dust suppressant, a blend of modified soybean protein isolate, carboxy methylcellulose for viscosity enhancement, and methanesiliconic acid sodium as a water-resistant additive. This suppressant works by exposing hydrophobic functional groups in its protein-surfactant composite, allowing coal dust to absorb these groups, and become cemented, thereby effectively suppressing dust. A recent study by Yang et al. (2024) demonstrated that natural cellulose (fiber), when chemisorbed onto the pyrite surface, masks the surface Fe^2+^ ions. This masking inhibits the reaction between pyrite and O_2_, consequently decreasing the production of •OH in the presence of molecular oxygen. The addition of calcite to iron-containing coals has been shown to reduce levels of BAI, a key factor contributing to the toxicity of mixed coal dust (Aladdin et al. 2013; Zhang Qi, Huang, 2005).

Dust particles can acquire varying electric charges after pulverization, which can affect electrical attraction or repulsion between dust particles and spray drops (Polat 1993; Tessum and Raynor 2017). Particle moisture content, composition, ambient humidity, and particle size are among the factors affecting the sign and magnitude of charges on the dust particles (Johnston et al. 1985). Chemical additives that carry electric charges, whether anionic or cationic, have the potential to neutralize charged dust particles. This neutralization can subsequently influence both the efficiency with which these particles are collected and their surface reactivity (Polat et al. 2000; Tessum et al. 2014; Tessum and Raynor 2017). A decreased negative surface charge of quartz by the adsorption of metallic cations has shown a decrease in the quartz hemolytic activity (Nolan et al. 1981).

Due to the complex surface chemistry of different minerals and coal, a single universal detoxification mechanism for respirable coal dust does not exist. Therefore, an in-depth investigation into the underlying chemistry and mechanisms of toxicity associated with minerals as well as coal in different coal ranks is essential. Many factors need to be considered in the context of coal dust toxicity, including water quality, coal rank, the synergistic or antagonistic effects of the composite phases, particle size and aging, as well as the potential hazards and costs of targeted chemical agents. Further investigation using simulated lung fluid solution is essential to determine how these fundamental experimental and theoretical insights would manifest in a human lung environment.

## Summary

With the increase in lung diseases related to respirable coal dust, such as CWP among U.S. coal miners in certain regions since the early 2000 s, additional investigations on physicochemical characteristics, surface properties, and mechanisms of surface oxidation for the different components of coal dust are warranted. Adverse health effects of respirable coal dust are partly linked to the toxicity of the constituent particles. In addition to fine size, the surface chemistry properties of the dust particles are also of great concern with regards to their potential to induce the formation of ROS such as •OH. Surface area, surface functional groups, metal content, hydrophobicity, surface charge, adsorption capacity, solubility, chemical composition, and pH are among the most important surface chemistry properties of the dust particles that can influence their •OH generation. The surface chemistry and biological activity of respirable coal particles are influenced by factors, including the rank of coal, oxygen and trace metal content, surface oxidation (aging), surface charge, the presence of organic aromatic compounds, iron content, carbon-centered coal radicals, organic sulfur, and EPFRs. There is a significant gap in understanding the role of coal rank in CWP and the relationship between coal rank and cell cytotoxicity. This gap highlights the need for further exploration of these issues from the perspective of surface chemistry. Pyrite-induced formation of •OH is affected by the oxidation and dissolution chemistry of pyrite. Coal-pyrite may exhibit remarkable differences in dissolution and electro-kinetic behavior, and surface morphology than ore-pyrite, which may result in more •OH generation by coal-pyrite. While not fully understood, silicon-based radicals like SiO• on the freshly fractured quartz surface are believed to cause the generation of •OH. In addition to direct reactivity of the fresh mineral surface, other properties including quartz origin, hydrophilicity, and the presence of metal impurities like iron and aluminum are also believed to contribute to the quartz-induced •OH and its pathogenic activity in biological systems. Clays associated with coal, particularly those containing iron, are significant for their ability to generate •OH. The surface chemistry dependence of •OH generation by coal dust components suggests that chemical reagents, when added to dust suppression systems, could alter dust surface properties, and mitigate the •OH formation. In this context, factors including water quality, as well as the potential hazards and costs of targeted chemicals, should be considered. Studies using simulated lung fluid would help determine how experimental and theoretical insights would manifest in a human lung environment.

## Data Availability

This is a review paper without data being generated.
